# On the Reduction of Subcoracoid Dislocation of the Humerus

**Published:** 1894-12

**Authors:** 


ON THE REDUCTION OF SUBCORACOID DISLOCATION OF THE HUMERUS. (*Intercolonial Quarterly Journal of Medicine and Surgery*, August, 1894.) By R. Hamilton Russell, F. R. C. S. (Eng.) of Melbourne, Victoria. The writer employs the following method: The patient being comfortably placed in a recumbent position, the surgeon lightly takes the wrist on the injured side, raises the arm justoff the bed, and pauses for a few seconds. He next proceeds with a movement so slow as to be almost imperceptible, to abduct the arm until it is extended above the shoulder at an angle of forty-five degrees with the axis of the trunk, but in the same plane. Two or three minutes should have been taken in this procedure, the movements having been made not only slowly, but perfectly steadily, remembering that any suddenness of movement will assuredly call forth muscular contraction in response; there must also have been, hitherto, an absence of traction on the arm Now, however, traction must be employed for the first time, but with the same insidiousness as to previous movements, and the humerus will be so easily drawn into the glenoid cavity, that both patient and surgeon may be deceived as to the reduction having taken place. The writer has had opportunity of trying this method in several cases, and has not yet failed to reduce the dislocation, without chloroform, and without inflicting the slightest pain on the patient. The writer considers the most appropriate designation would be to call this method reduction by "stealth." The author suggests that this plan might be tried in the rarer dislocations of the shoulder, and in dislocations of the hip.—*International Medical Magazine.*

